# Quantification of Dark Protein Populations in Fluorescent Proteins by Two-Color Coincidence Detection and Nanophotonic Manipulation

**DOI:** 10.1021/acs.jpcb.2c04627

**Published:** 2022-10-03

**Authors:** Gobert Heesink, Cécile Caron, Kirsten van Leijenhorst-Groener, Robert Molenaar, Theodorus W. J. Gadella, Mireille M. A. E. Claessens, Christian Blum

**Affiliations:** † Nanobiophysics (NBP), MESA+ Institute for Nanotechnology and Technical Medical Centre, Faculty of Science and Technology, University of Twente, P.O. Box 217, 7500 AEEnschede, The Netherlands; ‡ Section of Molecular Cytology, Swammerdam Institute for Life Sciences, University of Amsterdam, P.O. Box 94215, 1090 GEAmsterdam, The Netherlands

## Abstract

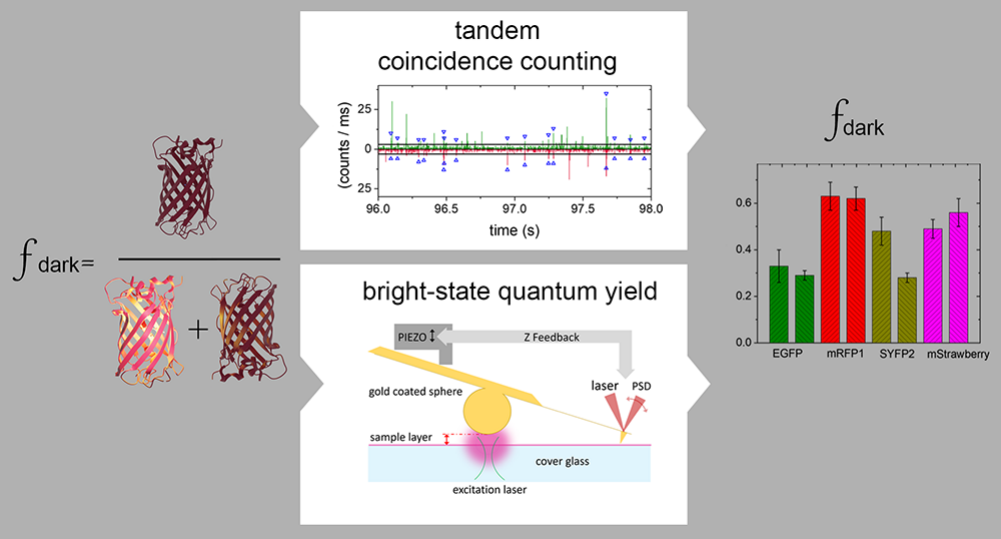

Genetically encoded visible fluorescent proteins (VFPs) are a key tool used to visualize cellular processes. However, compared to synthetic fluorophores, VFPs are photophysically complex. This photophysical complexity includes the presence of non-emitting, dark proteins within the ensemble of VFPs. Quantitative fluorescence microcopy approaches that rely on VFPs to obtain molecular insights are hampered by the presence of these dark proteins. To account for the presence of dark proteins, it is necessary to know the fraction of dark proteins (*f*_dark_) in the ensemble. To date, *f*_dark_ has rarely been quantified, and different methods to determine *f*_dark_ have not been compared. Here, we use and compare two different methods to determine the *f*_dark_ of four commonly used VFPs: EGFP, SYFP2, mStrawberry, and mRFP1. In the first, direct method, we make use of VFP tandems and single-molecule two-color coincidence detection (TCCD). The second method relies on comparing the bright state fluorescence quantum yield obtained by photonic manipulation to the ensemble-averaged fluorescence quantum yield of the VFP. Our results show that, although very different in nature, both methods are suitable to obtain *f*_dark_. Both methods show that all four VFPs contain a considerable fraction of dark proteins. We determine *f*_dark_ values between 30 and 60% for the different VFPs. The high values for *f*_dark_ of these commonly used VFPs highlight that *f*_dark_ has to be accounted for in quantitative microscopy and spectroscopy.

## Introduction

The use of genetically encodable visible fluorescent proteins (VFPs) is at the center of life science research since it can provide insights into cellular processes.^1−4^ VFPs have been used to report on the expression of proteins, to observe the subcellular localization of VFP-tagged proteins, to study interactions, and to observe conformational changes of proteins in cells.^5−9^ Photophysically, VFPs are much more complex than chemical dyes. A VFP ensemble behaves more like a complex fluorophore mixture than like an ensemble of identical emitters.^10−13^ This photophysical complexity may have consequences for the application of VFPs in quantitative microscopy and spectroscopy approaches.^14^ A major problem for many fluorescence microscopy and spectroscopy applications that rely on VFPs is the presence of dark proteins that contain no or non-emitting chromophores for which nonradiative decay is the dominating decay channel. Although they are difficult to observe directly, several studies show that VFP samples typically contain dark proteins.^15−18^ As a result of the presence of these dark proteins, fluorescence microscopy and spectroscopy approaches that rely on counting and quantifying generally underestimate the amount of VFPs. This is especially problematic in the increasingly popular single-molecule (SM) fluorescence approaches. One way to reason that dark proteins hamper quantification is found in SM counting experiments aimed at obtaining copy numbers of VFP-labeled proteins in the cell since proteins tagged with dark VFPs are not counted. Similar arguments hold for quantitative colocalization and approaches that use Förster resonance energy transfer (FRET) to determine bound fractions. The presence of dark proteins in the ensemble also causes an underestimation of the fluorescence quantum yield (QY), as determined with conventional averaging methods.^19−22^ The underestimation of the QY not only means that the individual emitting molecules are brighter than expected, but it also impairs quantitative FRET distance measurements. To relate the observed energy transfer efficiency to a distance, the Förster radius *R*_0_ is needed and derived from the QY. Clearly, the presence of dark VFPs is a problem for quantitative fluorescence microscopy and spectroscopy. Because dark proteins do not fluoresce, quantifying the fraction of dark VFPs is challenging and not yet well established.

Here, we use and compare two different methods to determine the fraction of dark proteins (i.e., the dark fraction *f*_dark_) of four commonly used VFPs: EGFP, SYFP2, mStrawberry, and mRFP1. One of the methods relies on single-molecule two-color coincidence detection (TCCD),^23−25^ using tandem proteins, constructed of spectrally different VFPs. The other method relies on comparing the bright state QY (QY_bright_) of the VFP to the ensemble-averaged QY obtained in conventional approaches.^20,21^ Both methods show that all four VFPs contain a considerable fraction of dark proteins. For SYFP2 in the tandem construct, we find a larger *f*_dark_ than for the method looking at the non-tandem SYFP2. The larger *f*_dark_ might be attributed to a difference in maturation behavior of the protein in the tandem construct. This highlights that it is necessary to keep in mind that VFPs are photophysically complex and small changes may affect the ratio between emitting and non-emitting VFPs. For the other three VFPs, the *f*_dark_ values determined agree well between both methods. We determine the *f*_dark_ to be ∼30% for EGFP, ∼50% for mStrawberry, and ∼60% for mRFP1 in both methods. This shows that both methods are suitable to obtain the dark fractions of VFPs, although they are very different in nature. We conclude that the dark fractions of the VFPs studied are considerable and need to be accounted for in quantitative microscopy and spectroscopy.

## Materials and Methods

### Construction and Expressions of Proteins and Protein Tandems

mStrawberry^26^ and EGFP^27^ were kind gifts from Roger Tsien and David Piston, respectively. For the production of the proteins SYFP2,^28^ mStrawberry, EGFP, and mRFP1 and the SYFP2–mStrawberry tandem,^29^ the respective vectors with a his-tag sequence for purification were cloned into a pRSETB expression system. To create the EGFP–mRFP1 vector, an EGFP-N1 vector (Clontech) was digested with AflII and NotI. Subsequently, a 26 bp linker was ligated in the EGFP-N1, creating a BamHI restriction site. The EGFP stop codon was removed by site-directed mutagenesis (QuikChange, Agilent). The mRFP1-pRSETB vector and EGFP-N1 vector with the inserted linker were digested with BamHI. The EGFP gene was isolated from an agarose gel and inserted into the mRFP1-pRSETB expression system with T4 DNA ligase (NEB). The obtained vector encodes for EGFP and mRFP1 connected by a QSGRLVPRDP linker.

The proteins were expressed in *E. coli* BL21(DE3) pLysS (Thermo Fisher) using kanamycin as the selective antibiotic. After induction of protein expression with 1 mM IPTG, the temperature was reduced from 37 to 20 °C for 4 h. Protein expression was continued overnight at 10 °C, after which the sample was pelleted. The cells were lysed in BugBuster master mix (Novagen) containing Pefabloc (Merck) as a protease inhibitor. All proteins were purified batchwise using a Ni-NTA agarose column (Qiagen) according the manufacturer’s protocol. Before storage, the buffer was exchanged to 10 mM Tris–HCl (pH 7.4) and 50 mM NaCl using PD-10 columns (Cytiva Life Sciences).

In the remainder of the manuscript, we will refer to SYFP2 and EGFP as protein A in the tandem (excited at 485 nm) and to mRFP1 and mStrawberry (excited at 560 nm) as protein B in the tandem.

### Recording TCCD Traces

Single-molecule burst analysis on the VFP tandem constructs was performed on a confocal microscope (PQ-MT200). The protein tandem solutions were diluted in a buffer of 10 mM Tris and 50 mM NaCl to approximately picomolar concentrations to obtain well-separated individual bursts in the confocal time traces; we will refer to these as burst traces. To excite the proteins in the tandems, pulsed interleaved excitation (PIE) of 485 and 560 nm was used (PicoQuant, LDH-485-D-C and LDH-D-TA-560B). The pulse rate of the two lasers used for excitation was 20 MHz. Pulses alternated between the two excitation wavelengths.

Excitation light was directed via an excitation dichroic mirror (Chroma, ZT488/561rpc-uf3) to the microscope’s objective (Olympus, UPLSAPO60XW 1.2 NA) and was focused to a diffraction-limited volume. Emission was collected by the same objective, confocally filtered by a 100 μm pinhole, and detected by two single-photon avalanche detectors (SPAD) (Excelitas, SPCM-AQRH-14-TR); emission was spectrally separated by a dichroic mirror (AHF, 560 LPXR) to create two spectrally separated detection channels. Emission was further restricted by bandpass filters, thus creating a “green channel” (Semrock, FF01-520/35) and an “orange channel” (Chroma, ET620/60x). Time tagging the detected photons from both channels allowed for assigning the respective excitation pulses to each detected photon in each channel. For each of the protein tandem samples, at least 16 time traces of 600 s each were recorded.

### Analysis of Single Protein Tandem Burst Traces

The time tagging and assignment of detected photons to the respective excitation wavelengths were used to create three data traces: (1) 485 nm excitation, emission in the green channel; (2) 485 nm excitation, emission in the orange channel; and (3) 560 nm excitation, emission in the orange channel. All data traces were binned using 1 ms bins. The binned time traces were corrected for background, donor leakage into the acceptor channel, and cross excitation, as described by Hellenkamp et al.^30^ After correction, bursts were identified based on a threshold of 3 photons/bin.

In a tandem of VFP A and VFP B, both A and B can be either emitting (bright) or non-emitting (dark). As a result, different combinations of emitting and non-emitting, dark proteins are possible: A_bright_–B_bright_, A_dark_–B_bright_, A_bright_–B_dark_, and A_dark_–B_dark_. To calculate *f*_dark_, the number of bursts containing the signature of the presence of both A and B (A_bright_–B_bright_), the number of bursts only containing the signature of A (A_bright_–B_dark_), and the number of bursts only containing the signature of B (A_dark_–B_bright_) need to be determined. We used the following criteria to identify if a burst contains A_bright_–B_bright_: simultaneous emission of A and B in a burst upon 485 and 560 nm excitation, respectively, or emission from B and/or A upon 485 nm excitation to account for FRET from A to B. A_bright_–B_dark_ and A_dark_–B_bright_ were identified by the absence of signature of A and B, respectively. The number of A_bright_–B_bright_, A_dark_–B_bright_, and A_bright_–B_dark_ burst events was counted.

Any burst that spans over three or more bins is considered to originate from a slowly diffusing species that is not of interest; these bursts are discarded.

### Determining the Dark Fraction from the Observed Events

The number of A_bright_–B_bright_ (*N*_bb_), A_bright_–B_dark_ (*N*_bd_), and A_dark_–B_bright_ (*N*_db_) can be expressed as a function of the dark fractions of protein A (*f*_dark,A_) and protein B (*f*_dark,B_), assuming that the occurrence of A_dark_ and B_dark_ in a tandem construct is independent. The total number of tandems diffusing through the detection volume is given as *N*_total_, yielding
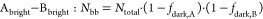

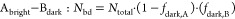

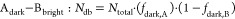
Rewriting these equations and solving the equations to *f*_dark,A_ and *f*_dark,B_ gives
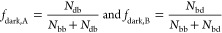


### Sample Preparation for LDOS Manipulation Measurements

The VFPs were stored in buffer (10 mM Tris, 50 mM NaCl, pH 7.4) and were diluted to concentrations between 0.5 and 1.5 μM in a 0.85% by weight aqueous solution of poly(vinyl alcohol) (PVA; Sigma-Aldrich, MW = 13,000–23,000). The VFP–PVA solution was spin-coated onto a microscopy cover slide (Menzel #1.5, 25 mm), resulting in a ∼15 nm thick film of PVA-embedded fluorophores. The film thickness and uniformity were verified by AFM.

### Fluorescence Lifetime and LDOS Manipulation

To observe the effect of the manipulation of the LDOS on the VFPs, we use a custom-built, TCSPC-based, confocal microscope (Olympus, IX71). For details, see refs (31, 32). In short, we excite the sample using a supercontinuum white light source (Fianium, SC-400-PP) operating at a repetition rate of 20 MHz. The excitation wavelengths of 488, 510, and 550 nm for EGFP, SYFP2, and mRFP1 and mStrawberry, respectively, were selected using an AOTF (Crystal Technologies, PC NI-VIS). Excitation light was passed through a single-mode fiber, collimated, and linearly polarized. Additional filters were used to further spectrally limit the excitation light: a 488/10 nm bandpass filter (Chroma, ZET488/10X), a 510/10 nm bandpass filter (Semrock, FF02-510/10-25), and a 556/20 nm bandpass filter (Semrock, FF01-556/10-25), respectively. The collimated light was focused into the sample by a microscope objective (Zeiss, C-Plan-Apochromat 63× NA1.4); the same objective was also used to collect the fluorescence. The fluorescence was spatially filtered by a pinhole and spectrally filtered to remove remaining excitation light. The used filters are as follows: a 550/88 nm bandpass filter for 488 nm excitation (Semrock, FF01-550/88-25), a 532 nm longpass filter for 510 nm excitation (Semrock, BLP01-532R-25), and a 590 nm longpass filter for 550 nm excitation (Olympus, BA590). An additional shortpass filter (Semrock, FF01-770/SP-25) was used in all experiments to suppress stray light from the AFM used for LDOS manipulation (see below). Photons were detected using an SPAD (MPD, PD1CTC), and photon arrival times were determined and registered relative to the excitation pulse using a TCSPC Counter Card (Becker & Hickl, SPC-830).

To control the LDOS of the VFPs in the PVA film, we change the distance between the VFPs and a metallic mirror. In the experiments, we used a gold-coated sphere (Duke Standards, 100 μm, coated with 3 nm of Cr and 100 nm of Au) glued to the base of an AFM cantilever as a metallic mirror. To control the LDOS, the distance between the mirror and the VFPs was controlled using the deflection of the in-contact AFM cantilever. Changes in the axial position of the mirror result in a different LDOS experienced by the fluorophores. The effect of decreasing the mirror-to-sample distance on the fluorescence lifetime of the VFP was monitored. LDOS-lifetime measurements were recorded every 6 to 8 nm, starting typically at 600 or 800 nm above the VFP layer and then approaching the layer. The fluorescence decays recorded for each mirror-to-sample distance, representing different LDOS values, were fitted to a single exponential to obtain the fluorescence lifetime.

The obtained lifetimes as a function of the sample-to-mirror distance were subsequently modeled using a description of the LDOS based on a multilayer model.^33^ To model our sample, we used a system consisting of four layers of different refractive indexes *n*: (1) a very thick glass substrate (*n* = 1.52); (2) a 15 nm thick PVA layer (*n* = 1.46); (3) an air layer (*n* = 1) of variable thickness, depending on the mirror-to-sample distance; and (4) a gold layer of 100 nm (*n* = 0.44 + 2.43i).^34^ The differences in LDOS that the VFPs experience as a result of different orientations are also accounted for in the model by introducing a parameter that represents the average orientation. We fit the experimentally obtained lifetime versus sample-to-mirror distance data to the model using the Nelder–Mead method to find the minimum error using three free fit parameters: the average fluorophore orientation of the VFPs in the PVA film, the radiative decay rate, and the nonradiative decay rate. The obtained decay rates are the decay rates in PVA that has a higher refractive index than water. To make our data comparable to the data obtained in aqueous solution, we translated the radiative decay rate using the Strickler–Berg relation. The Strickler–Berg relation allows to determine the change of the radiative decay rate due to a change in the refractive index. The validity of the Strickler–Berg relation for fluorescent proteins has been experimentally demonstrated in a systematic study by Suhling et al.^35^

## Results

**Figure 1:**
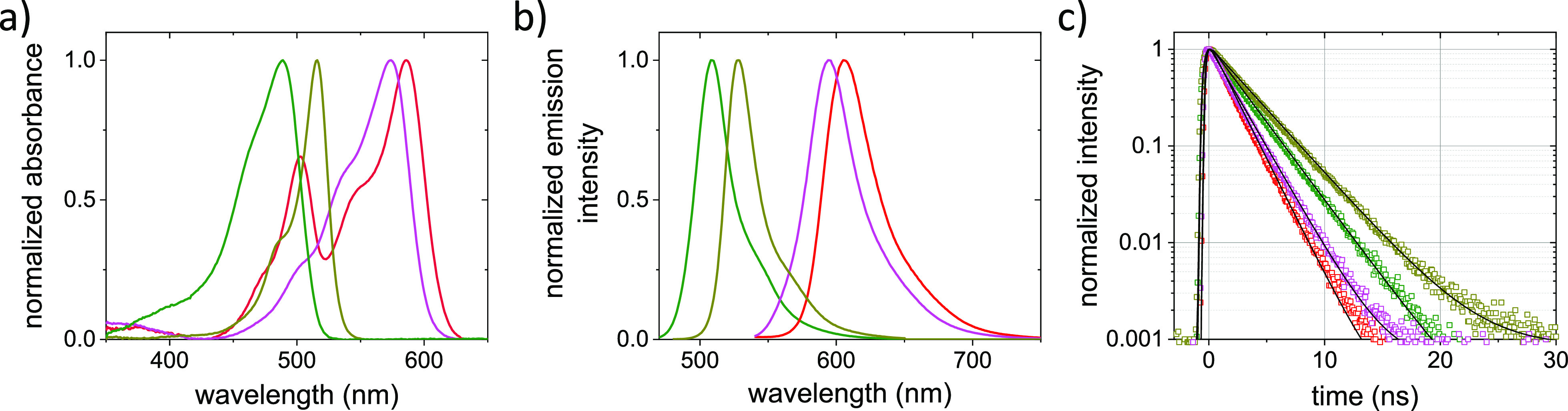
Photophysical properties of the VFPs. Data on EGFP are shown in green, SYFP2 in dark yellow, mStrawberry in magenta, and mRFP1 in red. (a) Absorbance spectra of the VFPs. The spectra are normalized to the maximal absorbance of the fluorophore. (b) Normalized fluorescence emission spectra of the VFPs. (c) Normalized fluorescence decays of the VFPs. For each protein, the data was fitted to a single exponential (black lines). From this fit, we determine the fluorescence lifetime of EGFP to be 2.7 ns, of SYFP2 to be 3.3 ns, of mStrawberry to be 2.1 ns, and of mRFP1 to be 1.8 ns.

To investigate the presence of dark proteins in VFPs, we selected four commonly used proteins. We choose EGFP and SYFP2 from the green/yellow emitting VFPs and mStrawberry and mRFP1 from the red emitting VFPs. The absorbance spectra, the emission spectra, and the fluorescence decays of the purified recombinantly expressed proteins are shown in Figure 1. The spectra of the green/yellow and the red emitting proteins are well separated (Figure 1a,b). The fluorescence decays of all the proteins can be fitted to a single exponential. The fit result for mRFP1 is slightly worse than for the other proteins, indicating the possible presence of a second decay component. Additional decay components for VFPs have been observed before and are attributed to the presence of emitting states of different brightness.^36^ In our analysis, such additional emitting states will complicate the analysis. However, the difference between the single and double exponential fits is minor. The decay of mRFP1 will therefore be treated as a single exponential in our analysis, like the decays of the other VFPs. From fitting the decays, we determine the fluorescence lifetime of EGFP to be 2.7 ns, of SYFP2 to be 3.3 ns, of mStrawberry to be 2.1 ns, and of mRFP1 to be 1.8 ns. The obtained fluorescence lifetimes agree well with the data found in the literature.^15,20,28,37^

**Figure 2:**
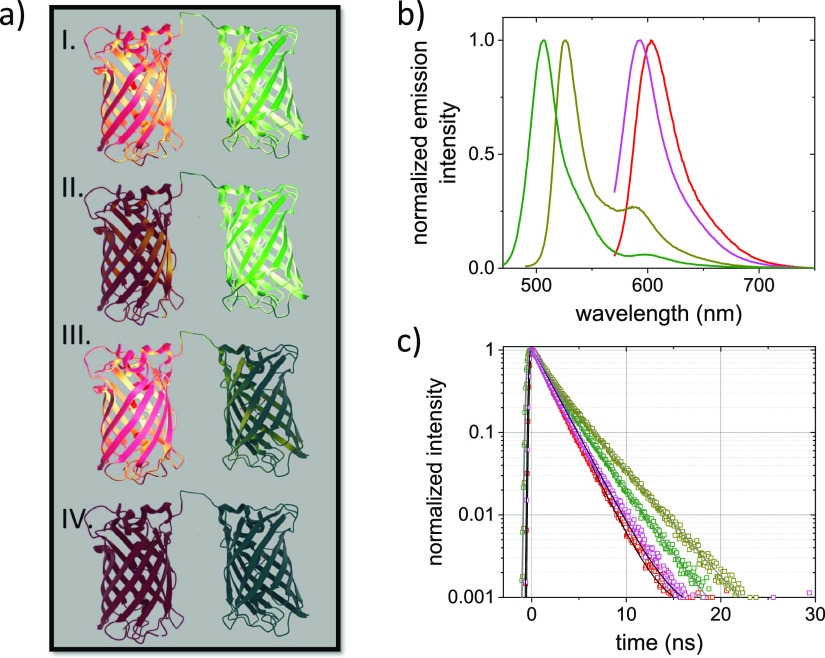
VFP tandems. (a) VFPs can either be emitting (bright) or non-emitting (dark). In a tandem of VFP A and VFP B, here shown in green and red, four different combinations of bright and dark proteins are possible. (I) A_bright_–B_bright_, (II) A_dark_–B_bright_, (III) A_bright_–B_dark_, and (IV) A_dark_–B_dark_. (b) Emission spectra of the VFP tandems excited at 450 nm. The EGFP–mRFP1 tandem is given in green, and the SYFP2–mStrawberry tandem in dark yellow. Emission from EGFP and SYFP2 is clearly visible. In addition, some emission from mRFP1 and mStrawberry can be observed as minor peaks around 600 nm. Excitation of the VFP tandems at 560 nm results in the emission of only mStrawberry (magenta) and mRFP1 (red). (c) Fluorescence decays of the VFP tandems excited at 485 nm are double exponential for EGFP (green) and SYFP2 (yellow). The double exponential decays consist of a fast component that we attribute to energy transfer from EGFP to mRFP1 (∼1.5 ns) and from SYFP2 to mStrawberry (∼0.7 ns). The slow decay components of 2.8 ns for EGFP and 3.3 ns for SYFP2 represent emission from the proteins in the ensemble that do not undergo FRET. Upon excitation at 560 nm, we observe the typical single exponential fluorescence decays with fluorescence lifetimes of 2.1 ns from mStrawberry (magenta) and 1.9 ns from mRFP1 (red).

To determine *f*_dark_, we start with TCCD to directly observe the fluorescence of individual tandems of VFPs. In a tandem of VFP A and VFP B, both A and B can be either emitting (bright) or non-emitting (dark). As a result, different combinations of emitting and dark proteins are possible: A_bright_–B_bright_, A_dark_–B_bright,_ A_bright_–B_dark_, and A_dark_–B_dark_ (Figure 2a). Interrogating the fluorescence from A and B at the single tandem level gives direct access to the occurrence of the different combinations and hence *f*_dark_ for both VFPs A and B (see Materials and Methods). To obtain the *f*_dark_ for the four selected VFPs, two tandems were constructed. The tandems consisted of EGFP–mRFP1 and SYFP2–mStrawberry, allowing for the individual excitation and detection of the two proteins in the tandem.

In Figure 2b, the emission spectra of the two produced tandems are presented. Upon excitation at 450 nm, strong peak emission of the EGFP and SYFP2 is observed at approximately 505 and 525 nm, respectively. In addition to the green/yellow fluorescence, we also observe some emission from mRFP1 and mStrawberry with peak intensities at approximately 600 and 590 nm, respectively. When the tandems are excited at 560 nm, only fluorescence from the red VFPs is observed. The fluorescence spectra of the red VFPs in the tandems agree with the spectra of the individual VFPs (Figure 1b). The fluorescence decays of the proteins in the tandems are shown in Figure 2c. Upon excitation at 560 nm, the fluorescence decays are identical to the decays for (non-tandem) mRFP1 and mStrawberry (Figure 1c). The fluorescence decays of the green/yellow proteins obtained after excitation at 485 nm change from single to double exponential decays compared to the data for the individual proteins presented in Figure 1c. The shorter lifetime component in this double exponential decay evidences that FRET occurs between the green/yellow and the red proteins. This is in agreement with the spectra shown in Figure 2a. The presence of the second, longer lifetime component shows that not all green/yellow VFPs in the ensemble undergo FRET. The flexible linker between the proteins allows for fast reorientation by diffusion and the averaging of the FRET efficiency. The absence of FRET in some of the tandem constructs is hence a first indication that some of the red VFPs are dark.

**Figure 3:**
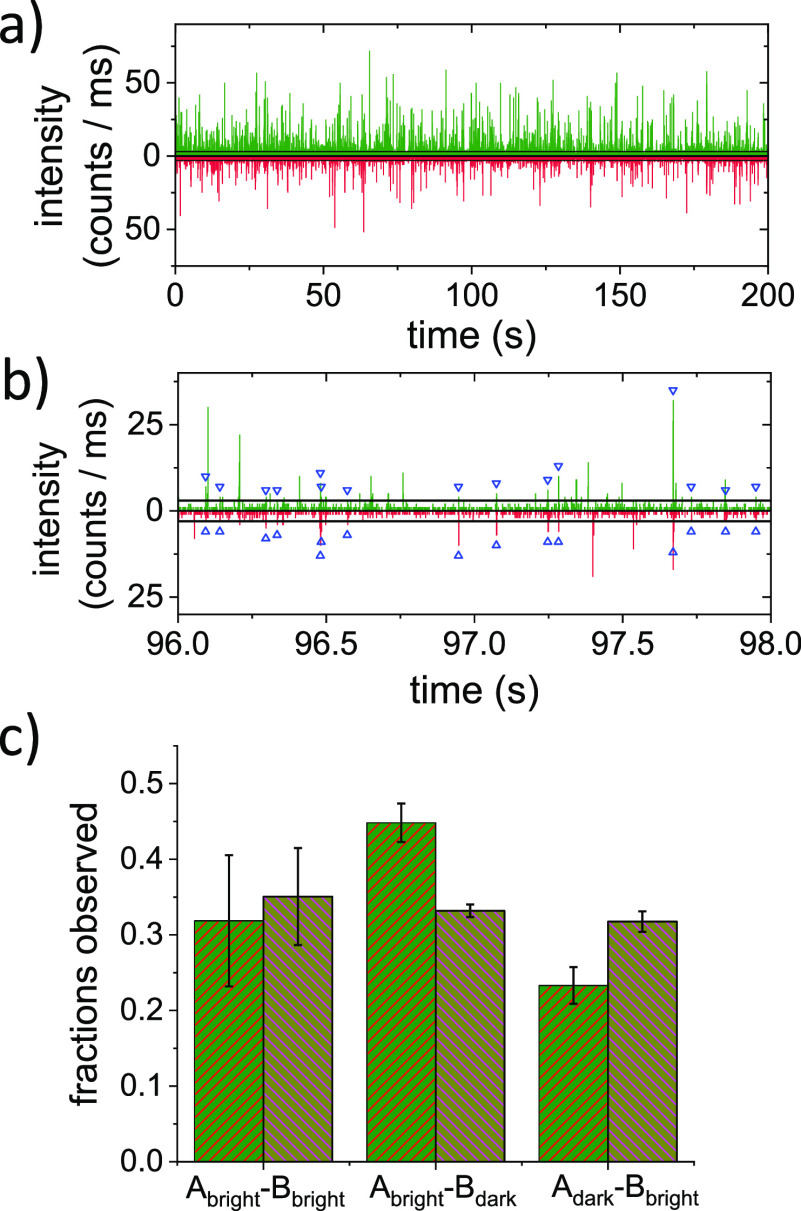
TCCD single tandem fluorescence bursts. (a) Typical fluorescence burst trace of the EGFP–mRFP1 tandem obtained upon PIE at 485 and 560 nm. The emission was split into two channels. In one channel, emission between 500 and 540 nm (shown in green) and in the other channel emission between 585 and 650 nm (shown in red) was detected. (b) Cutout of the trace shown in a. Individual bursts from single tandems diffusing through the focal volume can be clearly discriminated. To identify fluorescence bursts from the background, we use a threshold on both channels, as indicated by the horizontal lines shown in black. Coincident bursts originating from EGFP_bright_–mRFP1_bright_ are marked with blue triangles. The non-marked bursts make up the populations of EGFP_dark_–mRFP1_bright_ (red) and EGFP_bright_–mRFP1_dark_ (green) in the sample. (c) Quantification of the observed fractions of A_bright_–B_bright_, A_dark_–B_bright_, and A_bright_–B_dark_ for the EGFP–mRFP1 tandem (green hatched with red) and the SYFP2–mStrawberry tandem (dark yellow hatched with magenta).

In the next step, we analyze the emission of both the green/yellow and red proteins at the single tandem level. The samples were diluted to concentrations in the picomolar regime to ensure that on average ≪1 tandem is present in the confocal detection volume. In this detection volume, the red and green/yellow VFPs were excited individually at 560 and 485 nm respectively using PIE. The emission from the tandem proteins was spectrally separated and recorded. The diffusion of individual tandems through the focal volume results in a burst of emission. In Figure 3a,b, we show a typical burst trace obtained for the EGFP–mRFP1 tandem under PIE excitation. Photon bursts originating from the emission of EGFP as well as from mRFP1 are clearly visible. The individual bursts are analyzed to obtain the occurrence of EGFP_bright_–mRFP1_bright_, EGFP_dark_–mRFP1_bright,_ and EGFP_bright_–mRFP1_dark_. Bursts showing simultaneous EGFP and mRFP1 emission evidence the presence of both an emitting EGFP and mRFP1 chromophore (EGFP_bright_–mRFP1_bright_). The absence of the emission signature of one of the proteins, while the other one is emitting, evidences that the respective protein in the tandem does not contain an emitting fluorophore, the protein is dark (EGFP_dark_–mRFP1_bright_ and EGFP_bright_–mRFP1_dark_). In Figure 3c, we present the fractions found for EGFP_bright_–mRFP1_bright_, EGFP_dark_–mRFP1_bright_, and EGFP_bright_–mRFP1_dark_. The experiment was also performed for the second tandem consisting of SYFP2–mStrawberry. The data was analyzed as outlined above; the obtained fractions for SYFP2_bright_–mStrawberry_bright_, SYFP2_dark_–mStrawberry_bright_, and SYFP2_bright_–mStrawberry_dark_ are given in Figure 3c.

The observed occurrences subsequently allowed us to calculate back the *f*_dark_ for all four proteins (see Materials and Methods). For all VFPs, we find a considerable fraction of non-emitting proteins. We deduce that for EGFP *f*_dark_ is 33 ± 7%, for mRFP1 *f*_dark_ is 63 ± 6%, for SYFP2 *f*_dark_ is 48 ± 6%, and for mStrawberry *f*_dark_ is 49 ± 4%.

In a second method to determine the fraction of dark proteins, we rely on comparing the QY of the VFPs determined with conventional, averaging methods to the QY determined by considering only bright, emitting fluorophores. Conventional methods, including the Parker–Rees and integrating sphere approaches, do not discriminate between bright emitting and absorbing but non-emitting, dark, fluorophores.^38,39^ As a result, the QY determined with these methods is an average over the emitting and non-emitting fluorophores. The presence of dark fluorophores in the ensemble thus limits the QY that can be reached; the measured average QY is lower than that of the bright fluorophores. Recently, different approaches that allow for the determination of the QY of the bright fluorophores (QY_bright_) have been introduced. To determine QY_bright_ methods with which the photonic environment, represented by the local density of optical states (LDOS), is manipulated are used.^22,40−42^ By manipulating the LDOS, the fluorescence lifetime of the fluorophore changes. Analyzing the lifetime change with the change in the LDOS gives access to the radiative and nonradiative decay rates and thus the QY. Since only emitting fluorophores contribute to the fluorescence lifetime, the QY obtained with these methods represents only bright fluorophores, it is therefore QY_bright_. The fraction of dark proteins (*f*_dark_) can subsequently be obtained from the averaged QY obtained in conventional methods and QY_bright_:^21^
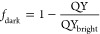
1

**Figure 4:**
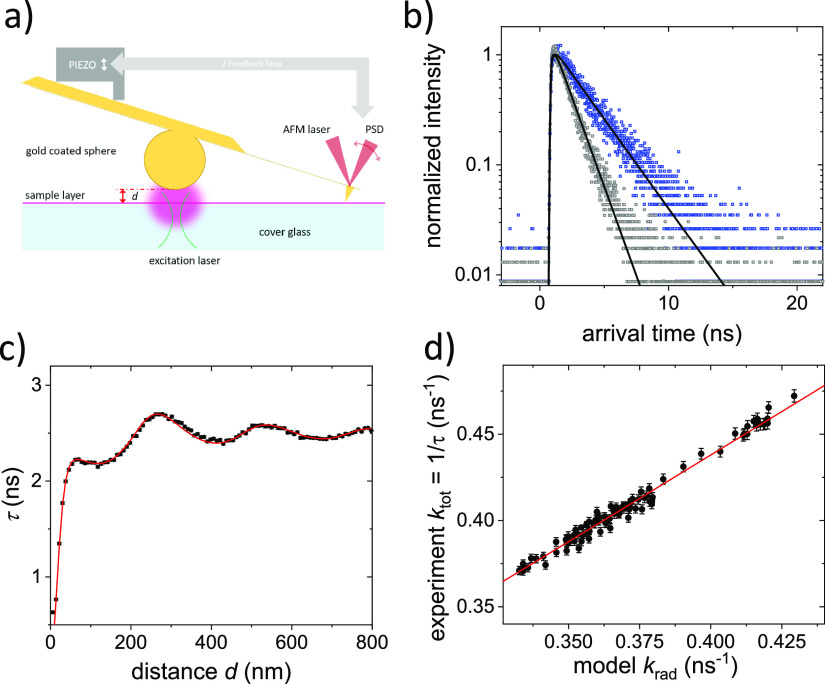
LDOS manipulation of VFPs. (a) Schematic of the method to control the mirror–sample distance and thus the LDOS the VFPs experience. The VFPs are embedded in a thin polymer film spin-coated onto a microscopy coverslip. A microcantilever to which a gold-coated sphere was attached serves as the LDOS manipulating probe. The cantilever is brought into contact with the coverslip, and the mirror-to-surface distance *d* is precisely controlled using the angular deflection of the microcantilever. (b) Fluorescence decay curves of EGFP recorded for two mirror-to-sample distances: *d* = 280 nm (blue) and *d* = 24 nm (gray). The decays are fitted with a single exponential (black lines), yielding fluorescence lifetimes of τ = 2.70 ns and τ = 1.35 ns, respectively. (c) The fluorescence lifetime of EGFP clearly shows the expected Drexhage oscillation with increasing distance to the mirror due to the modified LDOS. The experimentally obtained data (black squares) agrees very well with the fit to the multilayer model (red line). (d) Plot of the observed *k*_tot_ as a function of the modeled *k*_rad_ which is proportional to the LDOS. The red line represents a linear fit with a slope *k*_rad_ and intercept with the ordinate equal to *k*_nonrad_. We derive for EGFP a radiative decay rate of *k*_rad_ = 0.43 ns^–1^ in PVA and a nonradiative decay rate of *k*_nonrad_ = 0.038 ns^–1^.

In our experiments, we controlled the LDOS by placing the VFPs in front of a metallic mirror. The LDOS is dependent on the distance to the mirror and can hence be controlled. We precisely control the distance of the proteins to the mirror using AFM technology (Figure 4a).^43^ The proteins are immobilized in a thin PVA film. Since the fluorophore in the VFPs is completely encapsulated by the protein, the chemical environment that the fluorophore experiences remains unchanged by the immobilization. A 100 μm gold-coated sphere attached to the base of an AFM cantilever serves as the metallic mirror that is used to control the LDOS. Distance control between the mirror and the sample is realized by monitoring the deflection of the in-contact cantilever (Figure 4a). The VFPs on the coverslip under the mirror are excited confocally at 488, 510, and 550 nm for the green, yellow, and red VFPs, respectively. In our experiments, we vary the distance between the polymer-embedded VFPs and the mirror between 0 and 800 nm; fluorescence decays are measured using time-correlated single-photon counting (TCSPC) every 6 to 8 nm.

Each recorded fluorescence decay was fitted to a single exponential to obtain the fluorescence lifetime. No additional components were required to fit the data well. The fluorescence decay was observed to change with the distance to the mirror (Figure 4b). The fluorescence lifetime as a function of the distance to the mirror shows the expected oscillations (Figure 4c). To obtain QY_bright_, the data was modeled. Compared to the curvature of the mirror, the confocal spot is small, and the mirror was therefore modeled as a flat surface. The lifetime as a function of the mirror-to-sample distance was modeled using the description of the LDOS based on the multilayer system introduced by Chance et al.^33,44^ The changing lifetime as a result of the changing LDOS gives access to the radiative decay rate *k*_rad_ and the nonradiative decay rate *k*_nonrad_. We find very good agreement between the Chance model and the observed lifetimes. To determine the radiative and nonradiative rates, we parametrically plot the experimentally observed total decay rates *k*_tot_ (that is, the inverse of the lifetime τ) as a function of the calculated *k*_rad_ representing the LDOS at each emitter–mirror distance *d* (Figure 4d). We observe the expected linear relation with a slope equal to the radiative rate *k*_rad_ and an intercept with the ordinate equal to the nonradiative rate *k*_nonrad_ (see Figure 4d). To account for the higher refractive index of PVA compared to water, we use the Strickler–Berg relation^45^ to obtain *k*_rad_ in an aqueous environment. From the obtained *k*_rad_ and *k*_nonrad_, we calculate QY_bright_:
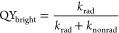
2

For EGFP, we find a QY_bright_ of 85 ± 4%. In a previous study, we reported EGFP to have a QY_bright_ of 72%.^22^ This 72% was based on a fit to only seven different LDOS data points. At that time, the mirror-to-sample distance was determined by a spacer layer, and each mirror-to sample distance had to be nanofabricated separately. Due to the limited amount of data points, it was not possible to consider the fluorophore orientation with respect to the mirror, the orientation was assumed to be isotropically distributed. The current data sets contain many more data points, which makes it possible to also take into account the fluorophore orientation in the fit. In contrast to our earlier assumption, we find that the fluorophore orientation is not isotropically distributed. The observed non-isotropic distribution of the fluorophore orientation in the PVA film is likely a result of (1) the limited film thickness relative to the size of the protein and (2) the spin-coating process. Taking into account the fluorophore orientation, we now obtain excellent fits to the LDOS data down to below 20 nm sample-to-mirror distance. For EGFP the averaged QY, which does not discriminate between bright and dark fluorophores, has been reported to be 60%.^46^ Using eq 2, we determine the fraction of dark, non-emitting, EGFP proteins to be 29 ± 2%.

**Figure 5:**
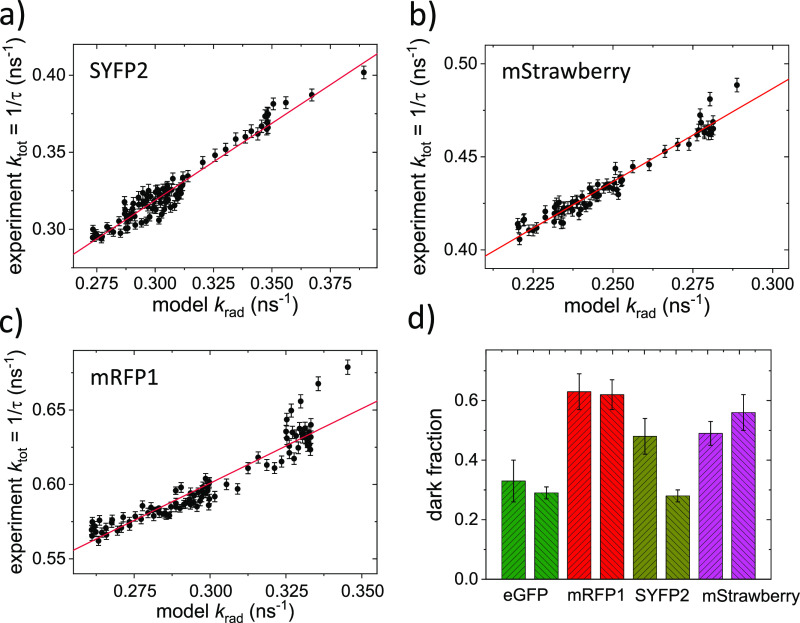
VFP dark fractions. Experimentally observed *k*_tot_ as a function of the modeled *k*_rad_. From the linear fit to the data (red line), we determine the decay rates. (a) For SYFP2, we determine a radiative decay rate of *k*_rad_ = 0.38 ns^–1^ in PVA and a nonradiative decay rate of *k*_nonrad_ = 0.017 ns^–1^. (b) For mStrawberry, we determine a radiative decay rate of *k*_rad_ = 0.31 ns^–1^ in PVA and a nonradiative decay rate of *k*_nonrad_ = 0.187 ns^–1^. (c) For mRFP1, we determine a radiative decay rate of *k*_rad_ = 0.37 ns^–1^ in PVA and a nonradiative decay rate of *k*_nonrad_ = 0.301 ns^–1^. (d) Dark fractions obtained from the two methods for the four VFPs studied. For each protein, the dark fraction obtained using the tandem burst analysis (hatched up) are shown left of the dark fraction obtained based on LDOS manipulation data (hatched down).

The other three VFPs were subjected to the same experimental and modeling procedures. For these proteins, the obtained QY_bright_ values are 95 ± 7% for SYFP2, 66 ± 7% for mStrawberry, and 62 ± 5% for mRFP1. Using the published QY based on averaging methods of 68% for SYFP2,^28^ 29% for mStrawberry,^26^ and 25% for mRFP1,^47^ we derive the fractions of dark proteins to be 28 ± 2% for SYFP2, 56 ± 6% for mStrawberry, and 59 ± 5% for mRFP1. For comparison, the *f*_dark_ values obtained for the different proteins from the burst analysis of protein tandems and from using LDOS manipulation are plotted in Figure 5d.

## Discussion

We determined the fraction of dark, non-emitting, fluorophores for four VFPs with two independent methods. The first method relies on the expression of spectrally different proteins in tandems. TCCD fluorescence spectroscopy experiments are subsequently performed on the single tandem level. The absence of one emitting fluorophore in the tandem can thus be directly observed in a two-color burst analysis. TCCD analysis of tandem constructs to determine dark fractions can even be performed in situ. A downside of the TCCD method is that for each protein (pair) of interest, a tandem has to be designed, expressed, and purified. In the second method, the *f*_dark_ of a VFP is determined using the QY_bright_ and the ensemble-averaged QY. QY_bright_ and ensemble averaged QY are both determined using normal (non-tandem) purified proteins that are anyway necessary for photophysical characterization. There are different methods that all rely on the modification of the LDOS to determine QY_bright_. With both methods, we detected a considerable fraction of dark fluorophores for all four VFPs tested. Generally, the *f*_dark_ of the green/yellow emitting VFPs is lower than that of the red emitting VFPs. This finding is in line with previous observations in which the apparent low QY of most red VFPs was attributed to an increased fraction of dark proteins.^21^

It is worth to note that blinking of VFPs has been observed. This blinking is a transition to a dark state induced by excitation light that spontaneously recovers to the emitting state. Such transitions will not contribute to the fraction of dark proteins determined using LDOS manipulation; however, they may add to the dark fraction determined by TCCD. The dark fractions determined by tandem burst analysis and the quantification method using LDOS manipulation are in good agreement for the *f*_dark_ of EGFP, mRFP1, and mStrawberry. The good agreement between the methods indicates that addition to the dark fraction due to blinking in the TCCD analysis plays no or only a minor role.

We obtain a *f*_dark_ of approximately 30% for EGFP with both methods, and this is in good agreement with the values reported earlier for mEGFP.^18^ The data on EGFP further supports that the QY_bright_ of this protein is indeed 85% and not 72% as we reported earlier based on a limited data set (see Discussionabove). This highlights that in determining QY_bright_, the orientation of the fluorophores in the polymer film has to be considered, assuming an isotropic orientation with respect to the mirror does not suffice for proteins embedded in thin polymer films.

For the red emitting mRFP1 protein, a very high *f*_dark_ of approximately 60% is found with both methods. A high *f*_dark_ is expected since mRFP1 is known to not easily mature. mRFP1 samples therefore contain a noticeable fraction of dark, green absorbing fluorophores. Quantitatively, the obtained *f*_dark_ for mRFP1 is in excellent agreement with an earlier study in which the dark fraction was determined to be 60% in a fluorescent tandem using fluorescence resonance energy transfer analysis.^16^

For mStrawberry, we find good agreement between the *f*_dark_ values obtained with both methods. The high *f*_dark_ of approximately 50% is typical for red emitting proteins and explains the low QY that is found using ensemble averaging methods.

For only one of the proteins, SYFP2, the *f*_dark_ obtained with TCCD on the tandem constructs is considerably higher than the *f*_dark_ obtained using LDOS modification on samples containing single, non-tandem SYFP2s. Since SYFP2 is acting as a FRET donor in the tandem, it is not possible to use the LDOS modification method to directly determine the QY_bright_ of SYFP2 in the tandem. Considering the good agreement between the *f*_dark_ values obtained with both methods for the other VFPs, this difference for SYFP2 is likely not a result of the method used. The LDOS data obtained for SYFP2 fits very well to the model. To fit the data, all fit parameters match expectations; this makes it unlikely that we underestimate the *f*_dark_ of SYFP2 with the LDOS method. The photophysical characterization of SYFP2 of the tandem also shows no deviation in the fluorescence SYFP2 spectrum or unexpected behavior in the fluorescence decay. One explanation for the observed high *f*_dark_ in the burst analysis is that SYFP2 maturated differently in the tandem constructs. Incomplete maturation of SYFP2 in the tandem might have resulted in an increased fraction of SYFP2 proteins that did not contain an emitting fluorophore. This highlights that it is necessary to keep in mind that VFPs are photophysically complex. Apparently, small changes in conditions may affect the ratio between emitting and non-emitting VFPs.
